# Supplementary material for: A methodology for psycho-biological assessment of stress in software engineering
Source: PeerJ Comput Sci. 2020 Aug 10;6:e286. doi: 10.7717/peerj-cs.286 (PMC7924460; doi:10.7717/peerj-cs.286)
Supplement: Supplemental Information 1 [file peerj-cs-06-286-s001.docx]

**Socio-demographic questionnaire**

**General questions**

**Date:**

**Last Name: First Name:**

**Gender:**

**Age: Date of Birth: Pseudonym:**

(Assigned at the day of the experiment)

**Occupation** (Students please state course of studies and semester)

**Please tick highest degree and current education**

**Total education (including study) itself:** Years: ___

Volksschule O; Hauptschule O; Realschule O; Fachabi O; Abi O; Studium O

**Education Father:**

Volksschule O; Hauptschule O; Realschule O; Fachabi O; Abi O; Studium O

**Education Mother:**

Volksschule O; Hauptschule O; Realschule O; Fachabi O; Abi O; Studium O

**How satisfied are you with the choice of study?**

🞎 very satisfied 🞎 satisfied 🞎 less satisfied 🞎 totaly dissatisfied

**What is your relationship status?**

🞎in relationship (unmarried) 🞎married and living together 🞎Single

**With which hand you perform most of the activities?**

🞎 left 🞎 right

**Do you have psychiatric or neurological conditions?**

🞎 no 🞎 yes

If so, which __________________________________________________
