# Supplementary material for: A methodology for psycho-biological assessment of stress in software engineering
Source: PeerJ Comput Sci. 2020 Aug 10;6:e286. doi: 10.7717/peerj-cs.286 (PMC7924460; doi:10.7717/peerj-cs.286)
Supplement: Supplemental Information 2 [file peerj-cs-06-286-s002.docx]

**PANAS**

Der Fragebogen enthält eine Reihe von Wörtern, die verschiedene Gefühle und Emotionen beschreiben. Lesen Sie jedes Wort und kreutzen Sie an wie stark Sie es jeweils empfinden. Beschreiben Sie damit, wie Sie sich während der letzten Minuten gefühlt haben.

|  | 1 | 2 | 3 | 4 | 5 |
| --- | --- | --- | --- | --- | --- |
|  | **gar nicht** | **ein wenig** | **mittel** | **ziemlich** | **extrem** |
| interessiert |  |  |  |  |  |
| bekümmert |  |  |  |  |  |
| angeregt |  |  |  |  |  |
| beunruhigt |  |  |  |  |  |
| stark |  |  |  |  |  |
| schuldig |  |  |  |  |  |
| erschreckt |  |  |  |  |  |
| feindselig |  |  |  |  |  |
| begeistert |  |  |  |  |  |
| stolz |  |  |  |  |  |
| reizbar |  |  |  |  |  |
| wachsam |  |  |  |  |  |
| beschämt |  |  |  |  |  |
| schwungvoll |  |  |  |  |  |
| nervös |  |  |  |  |  |
| entschlossen |  |  |  |  |  |
| aufmerksam ängstlich |  |  |  |  |  |
| ängstlich |  |  |  |  |  |
| aktiv |  |  |  |  |  |
| furchtsam |  |  |  |  |  |
| kompetent |  |  |  |  |  |
|  |  |  |  |  |  |
|  |  |  |  |  |  |
|  | 1 | 2 | 3 | 4 | 5 |
|  | **gar nicht** | **ein wenig** | **mittel** | **ziemlich** | **extrem** |
|  |  |  |  |  |  |
| Ärger |  |  |  |  |  |
| Ekel |  |  |  |  |  |
| Freude |  |  |  |  |  |
| Trauer |  |  |  |  |  |
| Überraschung |  |  |  |  |  |
| Furcht |  |  |  |  |  |
| Stress |  |  |  |  |  |

Wie schätzen Sie Ihre Kenntnisse ein? Bitte Kreutzen Sie ihre momentane Einschätzung an.

|  | **Sehr Schlecht** | **Schlecht** | **Mittel** | **Gut** | **Sehr Gut** |
| --- | --- | --- | --- | --- | --- |
| Java Kenntisse |  |  |  |  |  |
| Findbugs  Kenntnisse |  |  |  |  |  |
| Eclipse Kenntisse |  |  |  |  |  |

PANAS

The questionnaire contains a series of words that describe different feelings and emotions. Read each word and tick how hard you feel about it. Describe by it, how you felt during the last minutes.

|  | 1 | 2 | 3 | 4 | 5 |
| --- | --- | --- | --- | --- | --- |
|  | **Not at all** | **some** | **medium** | **quite** | **extreme** |
| interested |  |  |  |  |  |
| distressed |  |  |  |  |  |
| animated |  |  |  |  |  |
| worried |  |  |  |  |  |
| strong |  |  |  |  |  |
| guilty |  |  |  |  |  |
| frightend |  |  |  |  |  |
| hostile |  |  |  |  |  |
| excited |  |  |  |  |  |
| proud |  |  |  |  |  |
| irritable |  |  |  |  |  |
| alert |  |  |  |  |  |
| ashamed |  |  |  |  |  |
| energetic |  |  |  |  |  |
| nervous |  |  |  |  |  |
| deterined |  |  |  |  |  |
| attentivly |  |  |  |  |  |
| afraid |  |  |  |  |  |
| active |  |  |  |  |  |
| fearful |  |  |  |  |  |
| competently |  |  |  |  |  |

|  | 1 | 2 | 3 | 4 | 5 |
| --- | --- | --- | --- | --- | --- |
|  | **Not at all** | **some** | **medium** | **quite** | **extreme** |
|  |  |  |  |  |  |
| Anger |  |  |  |  |  |
| Disgust |  |  |  |  |  |
| Joy |  |  |  |  |  |
| Sorrow |  |  |  |  |  |
| Surprise |  |  |  |  |  |
| Fear |  |  |  |  |  |
| Stress |  |  |  |  |  |
