# Supplementary material for: A methodology for psycho-biological assessment of stress in software engineering
Source: PeerJ Comput Sci. 2020 Aug 10;6:e286. doi: 10.7717/peerj-cs.286 (PMC7924460; doi:10.7717/peerj-cs.286)
Supplement: Supplemental Information 3 [file peerj-cs-06-286-s003.docx]

Please rate the following statements on the basis of the given scale and tick as appropriate:

1. When problems arise, I find ways to carry through.

| Not True | Hardly true | Rather true | Exactly True |
| --- | --- | --- | --- |
|  |  |  |  |

1. I always succeed in solving difficult problems, if I try.

| Not True | Hardly true | Rather true | Exactly True |
| --- | --- | --- | --- |
|  |  |  |  |

1. It does not give me any difficulty to realize my intentions and goals.

| Not True | Hardly true | Rather true | Exactly True |
| --- | --- | --- | --- |
|  |  |  |  |

1. In unexpected situations I always know how to behave

| Not True | Hardly true | Rather true | Exactly True |
| --- | --- | --- | --- |
|  |  |  |  |

1. Even with surprising events, I believe that I can handle them well.

| Not True | Hardly true | Rather true | Exactly True |
| --- | --- | --- | --- |
|  |  |  |  |

1. I can easily face difficulties because I can always trust my abilities.

| Not True | Hardly true | Rather true | Exactly True |
| --- | --- | --- | --- |
|  |  |  |  |

1. Whatever happens, I'll be fine.

| Not True | Hardly true | Rather true | Exactly True |
| --- | --- | --- | --- |
|  |  |  |  |

1. For every problem I can find a solution.

| Not True | Hardly true | Rather true | Exactly True |
| --- | --- | --- | --- |
|  |  |  |  |

1. When a new thing comes to me, I know how to handle it.

| Not True | Hardly true | Rather true | Exactly True |
| --- | --- | --- | --- |
|  |  |  |  |

1. If a problem arises, I can do it on my own.

| Not True | Hardly true | Rather true | Exactly True |
| --- | --- | --- | --- |
|  |  |  |  |
